# Seed Priming with Phytohormones: An Effective Approach for the Mitigation of Abiotic Stress

**DOI:** 10.3390/plants10010037

**Published:** 2020-12-25

**Authors:** Mohammad Saidur Rhaman, Shahin Imran, Farjana Rauf, Mousumi Khatun, Carol C. Baskin, Yoshiyuki Murata, Mirza Hasanuzzaman

**Affiliations:** 1 Department of Seed Science and Technology, Bangladesh Agricultural University, Mymensingh 2202, Bangladesh; saidur69bau@gmail.com (M.S.R.); farjanaraufrakhi@gmail.com (F.R.); 2 Department of Bio-functional Chemistry, Okayama University, Okayama 700-8530, Japan; mou151bau@gmail.com (M.K.); muta@cc.okayama-u.ac.jp (Y.M.); 3 Department of Agronomy, Khulna Agricultural University, Khulna 9202, Bangladesh; shahinimran124@gmail.com; 4 Department of Biology, University of Kentucky, Lexington, KY 40546-0091, USA; ccbask0@uky.edu; 5 Department of Plant and Soil Sciences, University of Kentucky, Lexington, KY 40546-0091, USA; 6 Department of Agronomy, Faculty of Agriculture, Sher-e-Bangla Agricultural University, Dhaka 1207, Bangladesh

**Keywords:** abscisic acid, abiotic stress, auxin, phytohormones, salicylic acid, stress signaling

## Abstract

Plants are often exposed to abiotic stresses such as drought, salinity, heat, cold, and heavy metals that induce complex responses, which result in reduced growth as well as crop yield. Phytohormones are well known for their regulatory role in plant growth and development, and they serve as important chemical messengers, allowing plants to function during exposure to various stresses. Seed priming is a physiological technique involving seed hydration and drying to improve metabolic processes prior to germination, thereby increasing the percentage and rate of germination and improving seedling growth and crop yield under normal and various biotic and abiotic stresses. Seed priming allows plants to obtain an enhanced capacity for rapidly and effectively combating different stresses. Thus, seed priming with phytohormones has emerged as an important tool for mitigating the effects of abiotic stress. Therefore, this review discusses the potential role of priming with phytohormones to mitigate the harmful effects of abiotic stresses, possible mechanisms for how mitigation is accomplished, and roles of priming on the enhancement of crop production.

## 1. Introduction

Due to the consequences of global warming, crop production and productivity are hampered in many localities. Different environmental constraints, such as drought, salinity, heat, cold, and heavy metals, can seriously affect plant growth and development [1,2,3,4]. The early stages of plants, such as seed germination and seedling establishment, are susceptible to these environmental constraints [5,6]. In this era, much attention has been given to developing approaches to alleviate the constraints of abiotic stresses on seed germination. Different physiological and non-physiological techniques are available for enhancing seed germination as well as alleviating abiotic stresses. Seed priming is a low-cost and effective physiological and biochemical process that stimulates seed germination, enhances morphological parameters, and improves plant growth and development under abiotic stress [7,8,9,10,11].

Plant hormones are known as phytohormones or plant growth regulators (PGRs). Phytohormones are chemical molecules produced by plants and have important roles in regulating plant growth and development. Auxins (IAAs), cytokinins (CKs), gibberellins (GAs), abscisic acid (ABA), salicylic acid (SA), and ethylene (ET) are well-known phytohormones that are essential for plant growth and development [8,12]. Phytohormones function as important chemical messengers and modulate many cellular processes in plants, and they can coordinate different signaling pathways during exposure to abiotic stresses [13,14]. Several studies have reported that phytohormones can interact with each other and manage the physiology of plants exposed to different biotic and abiotic stresses [15,16,17,18].

**Figure 1:**
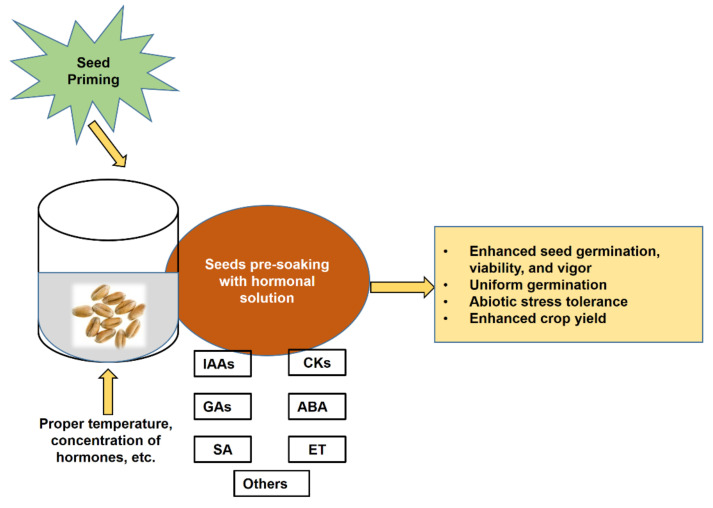
Schematic model showing possible effects of seed priming with phytohormones.

Seed priming with hormone solutions is referred to as hormonal priming, and hormonal seed priming plays an important role in seed metabolism [9]. Currently, hormonal seed priming is a commonly used technique to improve seed germination, seedling growth, and crop yield in adverse conditions [19,20,21]. Ensuring better germination and seedling vigor by seed priming would result in healthy and productive plants under adverse conditions (Figure 1).

In hormonal seed priming, seeds are pre-soaked with an optimal concentration of phytohormone, which enhances germination, seedling growth, and yield by increasing nutrient uptake through enhanced physiological activities and root production [22,23]. Seed priming with phytohormones has been studied in a range of crop species, and it modulates many physiological processes such as growth and development, respiration, and transpiration [24,25,26]. Phytohormones have a significant role in the biochemical, defense, and signaling pathways of plants [12]. Many researchers are working to develop effective approaches to alleviate abiotic stresses and enhance crop production. Seed priming with phytohormones can modulate the biochemical and molecular mechanisms making plants capable of tolerating these abiotic stresses, and these techniques are now very promising. Thus, the purpose of this review is to summarize the current understanding of the regulation of abiotic stresses through phytohormone priming and its future promise. Therefore, this review discusses hormonal seed priming and its role in stress mitigation, mechanisms of action of the hormones, and benefits for crop production in the future.

## 2. Commonly Used PGRs in Seed Priming

Among the plant growth regulators, IAAs, CKs, GAs, ABA, SA, and ET are commonly used in seed priming. In addition, methyl jasmonate (MeJA) and strigolactone have also been used in seed priming.

### 2.1. Auxin

**Figure 2:**
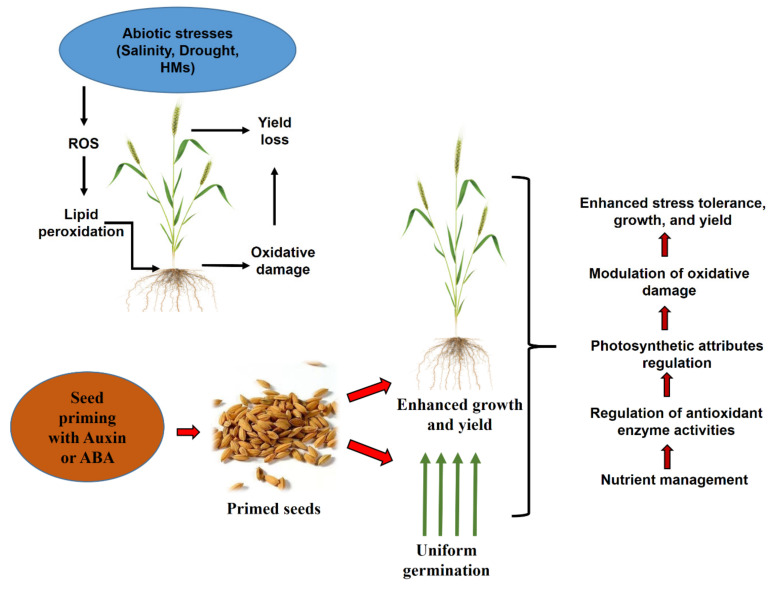
Proposed possible mechanisms used by auxin-and abscisic acid (ABA)-priming and their roles on the germination, growth, and development of plants under different stresses.

The IAAs were the first identified and are most well-known phytohormone that demonstrates a vital role in modulating plant growth and developmental processes such as root growth, cell elongation, vascular differentiation, and apical dominance [27,28]. IAAs promote plant growth not only under normal conditions but also under different stress conditions [29] (Figure 2). In higher plants, IAAs mainly exist in the form of IAA conjugates, and they are the primary free endogenous auxin involved in plant developmental processes such as lateral root formation. The exogenous application of IAAs induces the formation of adventitious roots [30,31].

Plant growth and development are hampered by different abiotic stresses, and seed priming with IAAs has been reported as an effective tool to reduce the effects of these stresses [23,32]. Seed priming with IAAs enhances cell division, photosynthetic activities, and translocation of carbohydrates, which results in lateral root initiation, flowering, and good stand establishment [33,34,35]. Seed priming with IAAs (1 ppm) enhanced the seedling establishment of *Bouteloua gracilis* [36], and in wheatgrass (*Agropyron elongatum*), seeds priming with IAAs at 50 ppm improved tolerance to drought stress by enhancing antioxidant enzyme activities such as catalase (CAT), superoxide dismutase (SOD), and peroxidase (POD) [7]. Under salinity stress, wheat seeds priming with IAAs (100, 150, and 200 mg L^−1^) regulated hormonal homeostasis, which enhanced the CO_2_ assimilation rate and ultimately resulted in increased grain yield [32]. Also, seed priming with IAAs improved the germination and growth of different species, such as rice (*Oryza Sativa*) and pigeon pea (*Cajanus cajan)*, under arsenic or cadmium (Cd) stress [26,37].

Iqbal and Ashraf [32] reported that seed priming with IAAs ameliorated salt stress via modulation of ion homeostasis in wheat and induced salicylic acid biosynthesis in leaves. Also, seed priming with IAAs activates calcium anion channels and inhibits K^+^ inward, rectifying channels during salt stress, which results in a reduction of transpirational water loss from plants. Modulation of the stomatal opening and closing helps plants reduce water loss via transpiration [38,39]. Consequently, plant growth is improved under stressful conditions. It is well known that exogenous and endogenous IAAs play an important role in stomatal movement and function as a positive regulator in the stomatal opening, but high concentrations of IAAs have a negative effect [40].

### 2.2. Cytokinin

CKs are the major plant hormones that regulate numerous aspects of plant growth and development, such as cell division, apical dominance, root formation, stomatal behavior, and chloroplast development [41,42]. It is well known that CKs application promotes crop production. For example, the application of CKs to cotton seedlings increased cotton yield by 5–10% [43]. CKs play an important role in plant pathogenesis, and CK application induced resistance against *Pseudomonas syringae* in *Arabidopsis thaliana* [44,45] and *Nicotiana tabacum* [45]. CKs may act as a biological agent to control diseases. For instance, *Pseudomonas fluorescens* G20-18 produces CKs, which controls *Pseudomonas syringae* infection in *Arabidopsis* and enhances biomass yield [46]. The exogenous application of CKs can mitigate the abiotic stresses on crop plants, which ultimately results in increased growth, development, and yield. Likewise, supplementation of CKs also reduces salinity stress in plants [47,48], and it increases starch accumulation in salt-stressed rice plants [49]. In addition, exogenously applied CKs increased net C-assimilation, net photosynthesis, and dry matter accumulation in *Epipremnum aureum,* which resulted in enhanced plant growth [50,51]. However, Zahir et al. [52] reported that exogenous application of CKs significantly increased the growth and yield of rice.

Seed priming with CKs or a combination of CKs and other plant hormones has resulted in the mitigation of abiotic stresses in various plant species (Table 1). Priming with CKs enhances chlorophyll (Chl) formation and biomass accumulation in plants, and it increases photosynthetic rate, promotes membrane stability, and maintains stable ionic levels. It has been reported that wheat seeds priming with kinetin (100 mg L^−1^, 150 mg L^−1^, and 200 mg L^−1^) enhanced germination and tolerance against salt by decreasing ABA and increasing IAAs concentrations [53]. Likewise, Mangena [54] reported that soybean seed priming with CKs (Benzyl adenine; 4.87 mg L^−1^) increased soybean root biomass, flowering, and fruiting under drought stress. Priming of aged groundnut (*Arachis hypogaea* L.) seeds with CKs (150 ppm) enhanced germination and seedling indices by enhancing antioxidant enzyme activities and decreasing oxidative damage [55]. However, the detailed mechanisms of how priming with CKs mitigate abiotic stress have not been investigated. CKs play a significant role in stomatal movement, and when applied exogenously, this PGR inhibits ABA-induced stomatal closure [56,57]. However, seed priming with CKs and its effects on stomatal movement are still unclear.

### 2.3. Gibberellin

GAs are plant growth hormones and have positive effects on seed germination, stem elongation, flowering initiation, and flower and fruit development [66,67]. GAs regulate plant growth and development during the entire life cycle of plants [68]. Demir et al. [68] reported that the application of GA_3_ significantly increased the germination speed of eggplant (*Solanum melongena*) seeds. Also, GAs can interact with other plant hormones and mediate many developmental processes in plants [69].

Different abiotic stresses, such as salinity, drought, chilling, heat, and heavy metals, inhibit proper nutrient uptake and photosynthesis, which ultimately results in stunted plant growth [70,71]. The exogenous application of GAs can mitigate abiotic stresses and enhance plant growth and development. The application of GAs in combination with poultry manure improved the growth of pepper (*Capsicum annuum*) plants and increased their salinity tolerance [72]. Moumita et al. [73] reported that exogenous application of GAs improved the growth of wheat (*Triticum aestivum*) plants and mitigated drought-induced oxidative damage by maintaining relative water content, balancing the antioxidant mechanism system, and conserving the Chl concentration. Foliar application of GA_3_ to tomato (*Solanum lycopersicum*) plants increased relative leaf water content, stomatal density, and Chl content by mitigating salinity stress [74]. Besides, GA_3_ stimulated plant growth and yield leaf of lettuce (*Lactuca sativa*) by enhancing biomass accumulation, leaf expansion, stomatal conductance, water use efficiency, and nitrogen use efficiency [75].

GAs are used as important seed priming agents to mitigate abiotic stresses in different crops (Table 2). Guangwu and Xuwen [76] reported that GAs (5 × 10^−5^ M) promoted seed respiration and lowered the ABA level and stimulated IAAs and GAs biosynthesis. In addition, wheat seeds treated with GA_3_ (100 mg L^−1^, 150 mg L^−1^, and 200 mg L^−1^) exhibited a decrease in the concentration of polyamines, ABA, and Na^+^ and an increase in the concentration of Ca^2+^ and K^+^ [23]. Moreover, wheat seeds primed with GAs (150 ppm) enhanced germination and seedling parameters under salt stress [77]. In the case of salt stress, maize seed priming with GAs (5 mg L^−1^) increased the shoot and root length and tissue water content [78]. Recently, Ma et al. [79] reported that seeds priming with GAs (50 µM) increased the germination rate, plant growth, and biomass production in *Leymus chinensis*. Likewise, seed priming with GAs increased the percentage and rate of seed germination and enhanced growth, yield, and yield-contributing characters of different crops species such as wheat, maize, and lentil [80,81,82,83]. However, more research is required to find the mechanisms of GA priming in abiotic stress mitigation.

### 2.4. Abscisic Acid

ABA is one of the major plant hormones and is also known as a stress hormone. It plays a vital role in mediating plant responses to various abiotic stresses, such as salt, heat, and drought [98,99,100]. ABA not only plays a role in abiotic stress mitigation but also plays a significant role in plant growth and development [101,102].

ABA is a potent seed priming hormone for the enhancement of germination as well as increased tolerance to various stresses by different crop species [103]. Rice seeds primed with ABA exhibited enhanced seedling growth and yield in saline soil by balancing nutrient uptake [103,104,105,106]. Likewise, priming rice seeds with ABA at 10 µM reduced alkaline stress by enhancing antioxidant enzyme activities and the activity of stress tolerance-related genes in the roots of rice seedlings [107]. Moreover, Wei et al. [108] reported that ABA (10 µM and 50 µM) priming of rice seeds improved the growth rate, survival rate, biomass accumulation, and root formation under alkaline stress. Also, seed priming with ABA enhanced salinity tolerance and increased the growth of rice, wheat, and sorghum [104,109]. Fricke et al. [110] reported that ABA priming promoted barley leaves growth by reducing transpirational water loss under saline conditions. Rice seeds primed with ABA at 10^−5^ M showed increased osmoregulation by reduced cellular Na concentration and increased proline and sugar accumulation in salt-stressed rice leaves [104]. The deterioration of *Agropyron elongatum* seeds was prevented by priming them with ABA at 50 ppm, which enhanced antioxidant enzyme activities [8]. Under saline soils, good stand establishment of sesame (*Sesamum indicum*) was achieved by ABA seed priming [111]. Zongshuai et al. [112] reported that the salt tolerance of wheat plants was enhanced by seed priming with ABA.

It has been reported that phytohormones are effective in the mitigation of heavy metal stress [12,26]. ABA biosynthetic gene expressions are induced by heavy metal stresses, which results in increased levels of endogenous ABA [12,18]. Under Cd stress, the germination of pigeon pea was improved by ABA (100 µM) priming [26]. However, the mechanism is still not clear, and the effects of seed priming with ABA on mitigation of heavy metal stress remain to be explored. Although seed priming with ABA enhances germination, many studies have reported that ABA inhibits seed germination which is dose dependent (10–30 µM) [113,114]. These differences may come from the endogenous and exogenous concentrations of ABA, whereas Srivastava et al. [115] reported the priming of mustard seeds with ABA (100 µM) increased the germination rate by 25% compared to the control under salt stress. In other words, an exogenous concentration may have an effect, and may enhance the germination at higher concentrations. However, how seed priming with ABA promotes germination needs more clarification with molecular studies.

ABA facilitates growth improvement via modulation of ion transport and regulation of stomatal movement in plants [116]. ABA is synthesized in plants under water-deficit conditions, and this induces stomatal closure via modulation of reactive oxygen species, reactive carbonyl species, cytosolic alkalization, and elevation of cytosolic calcium [38,117,118,119]. Exogenous application of ABA to plants also stimulates the regulation of stomatal movements, which helps reduce transpirational water loss. Marthandan et al. [120] reported priming *Arabidopsis* seeds with amino-butyric acid enhanced drought tolerance by accumulation of ABA and the closing of stomata. However, it is not known how seed priming with ABA helps regulate stomatal movements in plants. Based on information in the literature, we created a model showing how seed priming with ABA influences plant growth and development (Figure 2).

### 2.5. Salicylic Acid

SA is a phenolic plant hormone that regulates growth and development and many physiological processes, such as photosynthesis, respiration, transpiration, and the transportation of ions in plants. SA exhibits a key role in the activation, modulation, and regulation of numerous responses during exposure to abiotic and biotic stresses [102,121,122,123]. It is well known that SA generates a cascade of signaling pathways by interacting with other plant hormones such as ABA, MeJA, and ET and plays an important role in mitigating plant stresses [124,125]. Also, plant resistance to salinity, heat, and cell death under various hypersensitive stresses can be regulated by the presence of SA [126,127]. The exogenous application of SA enhanced maize (*Z. mays*) productivity under low temperature stress, as well as the germination and growth parameters of garden cress (*Lepidium sativum*) seedlings under salinity stress [128], and mitigated drought stress and enhanced the vegetative growth of safflower (*Carthamus tinctorius*) [129].

Seed priming with SA mitigates the effects of abiotic stresses and enhances yield in a range of crop species (Table 3).

**Figure 3:**
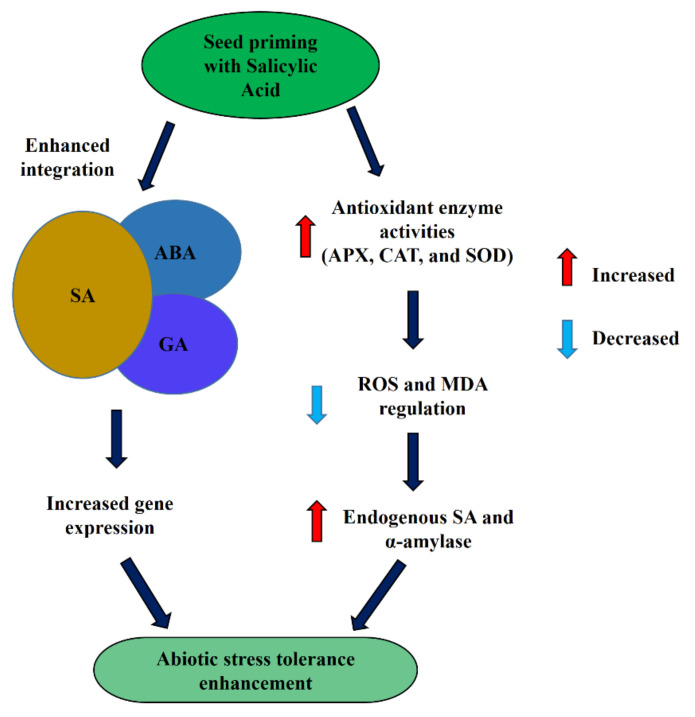
Mechanisms of SA priming for abiotic stress tolerance enhancement.

Seed priming with SA mitigated abiotic stresses by enhancing antioxidant enzyme activities such as CAT, APX, and SOD and regulating lipid peroxidation and H_2_O_2_ production. Likewise, seed priming with SA also increased the production of osmolytes such as proline and glycine betaine, which play an important role in mitigating different stresses [12,37]. Ion homeostasis and nutrient uptake were regulated by priming with SA at 0.25 mM and 0.50 mM, which enhanced the tolerance of heavy metal stress [130]. In addition, priming with SA at 0.5 mM also enhanced endogenous SA content and α-amylase activity during abiotic stresses [134]. Moreover, priming with SA enhanced the integration affinity among the phytohormones as a result of the GAs biosynthetic gene, and ABA catabolism gene expression enhanced and upregulated the GAs and ABA signaling pathways (Figure 3). In addition to abiotic stress mitigation, priming with SA has an important role in enhancing seed germination and crop productivity. Priming with SA at 100 mg L^−1^ enhanced emergence and early seedling growth in cucumber [151] and increased germination and productivity of *Vicia faba* [152] and sesame [153]. It has been reported that rice seeds primed with SA (100 ppm) had increased germination and accelerated seedling growth by ion absorption in PEG-induced water stress [131]. In addition, priming of rice seeds with SA enhanced tolerance to chilling stress by enhancing antioxidant enzyme activities and reducing oxidative damage [132]. SA induced stomatal closure and reduced transpirational water loss from plants [154,155]. Seed priming with SA has a role in the stomatal movement that has not been analyzed, and integration mechanisms with other phytohormones are still unclear.

### 2.6. Ethylene

The hydrocarbon ET is an important plant hormone and it is widely used for ripening fruits [156]. For example, the application of 100 μL L^−1^ of ET for 12 h stimulated the production of 1-amino cyclopropane-1-carboxylic acid (ACC: an ethylene precursor) and increased ACC oxidase activity, which accelerated the ripening of ‘Ataulfo’ mangoes [157]. The exogenous application of 5 mL L^−1^ ET improved the activity of CAT, APX, and SOD and reduced the activity of polyphenol oxidase (PPO) and POX, which prevented browning of the peel of the ‘Huangguan’ Pear (*Pyrus bretschneideri* Rehd cv. Huangguan) [158]. The exogenous application of ethephon (source of ethylene) to soybean (*Glycine max*) plants mitigated waterlogging stress by promoting the initiation of adventitious roots and by increasing root surface area, expression of glutathione transferases, and relative glutathione activity [159].

The exogenous application of ET has been an important player in the mitigation of abiotic stresses, but seed priming with ET has received little research attention. Nascimento et al. [160] reported that priming lettuce seeds with ACC increased germination at a high temperature (36 °C). Priming pigeon pea seeds with 10 mM ET (chloroethylphosphonic acid) increased the germination percentage under Cd stress conditions [26]. The combined application of ethephon and gibberellic acid to rice seeds increased α-amylase activity and sugar content [89]. Manoharlal and Saiprasad [161] reported that priming with ethephon improved the germination of soybean seeds. Further research is necessary to determine the effects of priming seed with ethylene on germination under different abiotic stresses.

### 2.7. Others

Jasmonic acid derivatives are widely used as a priming agent to ameliorate abiotic stresses. It has been reported that, with rice seed priming with MeJA at 2.5 mM and 5 mM, seeds experienced increased Chl content and photochemical efficiency under PEG stress [162]. Likewise, priming with MeJA improved the growth of broccoli sprouts under salinity stress [163]. In addition, priming with MeJA (1 mM) may function as a biocontrol agent and protect tomato seedlings against *fusarium* wilt [164]. Another phytohormone, brassinosteroids, has also been used as a priming agent and has been known to regulate plant growth and development and resistance to abiotic stresses [165]. It has been reported that the seed priming of lucerne (*Medicago sativa* L.) with brassinolide (5 µM L^−1^) improved seed germination and seedling growth under salinity stress [166]. Likewise, peanut seed priming with brassinosteroids at 0.15 ppm improved drought tolerance and increased the yield of peanut [167]. However, more research is necessary to find out the effects of seed priming with jasmonic acid and brassinosteroids under abiotic stresses.

## 3. Conclusions with Future Perspectives

Seed priming with phytohormones has emerged as a promising strategy in modern stress management as it protects plants against various abiotic stresses by increasing level of antioxidant enzyme activity, decreasing oxidative damage, and enhancing plant growth. Thus, seed priming with phytohormones improves the tolerance of crop plants to abiotic stress, and this technique can be utilized to maintain sustainable crop production in drought-, saline-, and flood-prone areas of the world. Seed priming with phytohormones not only improves the tolerance to abiotic stresses but also ensures hermonized germination by breaking the dormancy and enhancing viability. This review provides insight into the role of seed priming with phytohormones in mitigating the effects of abiotic stress on seed germination and plant growth. The data compiled in this review can be used for developing further extensive research on abiotic stress mitigation by seed priming with phytohormones. Seed priming with phytohormones has emerged as an effective seed treating tool for many crops, but treating conditions and methods differ from crop to crop, and seed priming with phytohormones has still limitations. For instance, prolonged seed treatment with hormonal solution during priming may cause the loss of seed tolerance to desiccation, which reduces seed viability. However, more research at the molecular level is required to clarify the mechanisms of involvement of phytohormones in seed priming, especially in the application methods, and phytohormones cross-talk and stress-responsive genes.

## Figures and Tables

**Table 1:** Seed-priming with cytokinin adopted for developing abiotic stress tolerance in plants.

Plant	Stresses	Responses of Plant	References
Soybean (*Glycine max*)	Drought	Improved drought tolerance in soybean plants	[54]
Pigeon pea (*Cajanus cajan*)	Salt	Prevented the damage caused by the apparatus involved in protein synthesis	[58]
	Cadmium	Tolerance to the effects of Cd stress	[26]
Basil (*Ocimum basilicum*)	Drought	Reduced negative effects of drought stress	[59]
Wheat (*Triticum aestivum*)	Salt	Decreased ABA concentration, increased IAAs concentration, and enhancement of salt tolerance	[60]
	Salt	Improved photosynthetic rate, water use efficiency and stomatal conductance, decreased Na^+^ and Cl^−^ level, increased K^+^ level	[61]
	Salt	Decreased electrolyte leakage and conferred salt tolerance	[62]
	Salt	Increased tissue N content and nitrate reductase activity	[63]
	Salt	Induced reduction in inorganic ion accumulation and increasing membranes stability and K^+^/Na^+^ ratio, enhanced chlorophyll formation and soluble sugar accumulation	[64]
	Salt	Alleviated salt stress by enhanced ethylene production	[65]

**Table 2:** Seed-priming with gibberellin and response of plant species.

Plants	Stresses	Responses of Plant	References
Pigeon pea (*Cajanus cajan*)	Cadmium	Increased germination speed index and germination percentage and tolerance to Cd stress	[26]
Pot marigold and Sweet fennel	Salt	Increased dry matter and enhanced tolerance to salinity by enhancing antioxidant enzyme activities	[84]
Milk Thistle (*Silybum marianum*)	Salt	Increased α-amylase activity and alleviated salt stress effects	[85]
Chickpea (*Cicer arietinum*)	Drought	Increased relative water content, seed protein, and reduced electrolyte leakage	[86]
Wheat (*Triticum aestivum)*	Salt	Promoted better salinity tolerance	[77]
Sorghum (*Sorghum bicolor*)	Drought	Increased CAT and APX activities	[87]
Corn (*Zea mays*)	Salt	Increased tissue water content	[78]
Maize (*Zea mays*), Pea (*Pisum sativum*), Grass pea (*Lathyrus sativus)*	Salt	Alleviated salt stress effects	[88]
Rice (*Oryza sativa*)	Flood	Increased α-Amylase activity, sucrose, glucose, and fructose content in seeds.	[89]
Alfalfa (*Medicago sativa*)	Salt	Induced enzymatic activities (SOD, CAT, GPX, APX, GR), and decreased lipid peroxidation, and reduced membrane damage of alfalfa.	[90]
Sponge gourd (*Luffa aegyptiaca*)	Salt	Prevented the adverse effect of salinity	[91]
Soybean (*Glycine max*)	Saline-alkali	Increased activities of the antioxidant defense system, photosynthetic pigment contents, better membrane integrity	[92]
Maize (*Zea mays*)	Salt	Reduced negative effect of salt stress	[93]
Sweet sorghum (*Sorghum bicolor*)	Salt	Enhanced water absorption and improved salinity tolerance	[94]
Maize (*Zea mays*)	Drought	Increased chlorophyll content and enhance drought tolerance	[95]
Okra (*Abelmoschus esculentus*)	Salt	Increased water content of the okra seedlings	[96]
Triticale	Salt	Reduced Na^+^ accumulation and increased K^+^ uptake	[97]

**Table 3:** Seed priming with salicylic acid (SA) and response of plant species.

Crops	Stresses	Responses of Plants	References
Rice (*Oryza sativa*)	Chromium	Increased chlorophyll content and proper nutrient uptake	[130]
	Water deficit	Decreased water stress	[131]
	Chilling	Enhanced antioxidant enzyme activities, detoxified ROS	[132]
	Salinity	Improved Na^+^/K^+^ and maintaining membrane integrity	[133]
Safflower (*Carthamus tinctorius*)	Drought	Enhanced antioxidant enzyme activities and reduced oxidative damage	[129]
Maize (*Zea mays*)	Chilling	Increased α-amylase and antioxidant enzyme activities and endogenous SA content	[134]
	Chilling	Enhanced enzymatic antioxidant activities, high tissue water content	[135]
	Lead	Increased glycine betaine and nitric oxide content and regulation of gene expression	[136]
	Chromium and UV-B	Reduced the accumulation of chromium and ROS	[137]
Wheat (*Triticum aestivum*)	Salinity	Decreased the electrolyte leakage	[138]
	Drought	Balanced nutrient uptake	[139]
	Osmotic	Resistance to osmotic stress	[140]
	Salinity	Higher contents of photosynthetic pigments, soluble sugar, and protein	[141]
	Boron toxicity	Increased photosynthetic pigments	[142]
	Cadmium	Modulates nutrient relations and photosynthetic attributes	[143]
Smooth vetch (*Vicia dasycarpa*)	Water deficit	Higher accumulation of proline and glycine betaine	[144]
Okra (*Abelmoschus esculentus*)	Chilling	Enhanced antioxidant enzyme activities and membrane integrity	[145]
Sorghum (*Sorghum bicolor*)	Drought	Improved antioxidant defense system	[146]
Tomato (*Solanum lycopersicum*)	Salinity	Decreased salinity stress	[147]
	Heat	Increased lycopene content	[148]
Pumpkin	Salinity	Protein contents and nitrate reductase were increased	[149]
Faba bean (*Vicia faba*)	Salinity	Higher osmotic solute content, carotenoids, and antioxidant enzyme activity	[150]
